# Cinnamic Acid Analogs as Intervention Catalysts for Overcoming Antifungal Tolerance

**DOI:** 10.3390/molecules22101783

**Published:** 2017-10-21

**Authors:** Jong H. Kim, Kathleen L. Chan, Luisa W. Cheng

**Affiliations:** Foodborne Toxin Detection and Prevention Research Unit, Western Regional Research Center, USDA-ARS, 800 Buchanan St., Albany, CA 94710, USA; kathy.chan@ars.usda.gov (K.L.C.); luisa.cheng@ars.usda.gov (L.W.C.)

**Keywords:** antifungal, antioxidant system, caspofungin, cell wall integrity, chemosensitization, cinnamic acids, intervention catalysts, small molecules, synergism

## Abstract

Disruption of fungal cell wall should be an effective intervention strategy. However, the cell wall-disrupting echinocandin drugs, such as caspofungin (CAS), cannot exterminate filamentous fungal pathogens during treatment. For potency improvement of cell wall-disrupting agents (CAS, octyl gallate (OG)), antifungal efficacy of thirty-three cinnamic acid derivatives was investigated against *Saccharomyces cerevisiae* *slt2*Δ, *bck1*Δ, mutants of the mitogen-activated protein kinase (MAPK), and MAPK kinase kinase, respectively, in cell wall integrity system, and *glr1*Δ, mutant of CAS-responsive glutathione reductase. Cell wall mutants were highly susceptible to four cinnamic acids (4-chloro-α-methyl-, 4-methoxy-, 4-methyl-, 3-methylcinnamic acids), where 4-chloro-α-methyl- and 4-methylcinnamic acids possessed the highest activity. Structure-activity relationship revealed that 4-methylcinnamic acid, the deoxygenated structure of 4-methoxycinnamic acid, overcame tolerance of *glr1*Δ to 4-methoxycinnamic acid, indicating the significance of *para* substitution of methyl moiety for effective fungal control. The potential of compounds as chemosensitizers (intervention catalysts) to cell wall disruptants (viz., 4-chloro-α-methyl- or 4-methylcinnamic acids + CAS or OG) was assessed according to Clinical Laboratory Standards Institute M38-A. Synergistic chemosensitization greatly lowers minimum inhibitory concentrations of the co-administered drug/agents. 4-Chloro-α-methylcinnamic acid further overcame fludioxonil tolerance of *Aspergillus fumigatus* antioxidant MAPK mutants (*sakA*Δ, *mpkC*Δ). Collectively, 4-chloro-α-methyl- and 4-methylcinnamic acids possess chemosensitizing capability to augment antifungal efficacy of conventional drug/agents, thus could be developed as target-based (i.e., cell wall disruption) intervention catalysts.

## 1. Introduction

**Figure 1:**
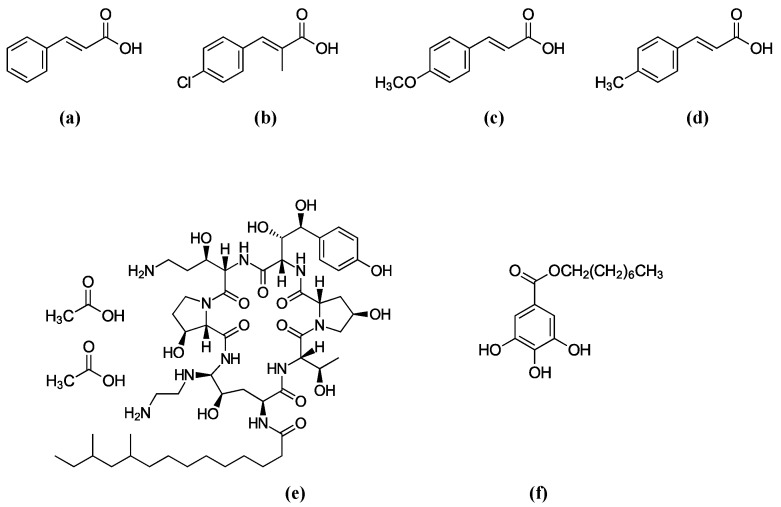
Structures of antifungal agents used in this study. (**a**) Cinnamic acid (Basic structure), (**b**) 4-Chloro-α-methylcinnamic acid, (**c**) 4-Methoxycinnamic acid, (**d**) 4-Methylcinnamic acid, (**e**) Caspofungin (CAS), (**f**) Octyl gallate (OG).

Mycotic diseases, such as human invasive aspergillosis caused by *Aspergillus* (e.g., *Aspergillus fumigatus*, *Aspergillus flavus*), or mycotoxin contamination by filamentous fungal pathogens (e.g., *Aspergillus parasiticus*—producing hepato-carcinogenic aflatoxins) are problematic since effective antifungal agents, especially those for control of drug/fungicide-resistant pathogens, are often very limited. Studies have shown that the fungal cell wall plays an important role during host infection, and thus has long been a target of many antifungal agents [1]. Currently, the lipopeptide drug echinocandins (e.g., caspofungin (CAS; Figure 1), micafungin, anidulafungin) are clinically applied to target the cell wall integrity system of fungal pathogens [2]. However, due to increasing concerns about the safety of certain antifungal drugs that have been in wide use and the impact of repeated exposure to these compounds on health and fungal resistance, industries constantly seek new antifungals or drug potentiators (viz., intervention catalysts) with improved health and safety profiles.

The cell wall integrity pathway is well characterized in the model fungus *Saccharomyces cerevisiae*, where the upstream mitogen-activated protein kinase (MAPK) signaling cascades (required for cell wall maintenance) are controlled by the enzyme protein kinase C [3]. Genome and functional comparisons further revealed that many genes in the cell wall integrity system in fungi, such as *Aspergillus nidulans*, *Aspergillus oryzae*, *A. fumigatus*, and *S. cerevisiae*, are well conserved [4,5]. For example, *A. nidulans mpkA*Δ (cell wall integrity MAPK mutant) exhibited hypersensitivity to the cell wall interfering agent calcofluor white (CW) [5]. Similar hypersensitive response to CW was also observed in *S. cerevisiae slt2*Δ, the orthologous cell wall integrity MAPK mutant of the model yeast [5].

Noteworthy is that the other signaling cascade, namely “antioxidant” MAPK pathway, is also involved in fungal susceptibility to cell wall disruptants [6,7,8]. In general, intact antioxidant MAPK pathway is necessary for the effectiveness of cell wall disruptants, while MAPK mutations cause drug resistance [6,7,8]. For example, *S. cerevisiae* antioxidant MAPK mutants, e.g., *hog1* (MAPK) or *pbs2* (MAPK kinase; MAPKK), were tolerant to cell wall-disrupting agents [6,7,8]. Furthermore, mutants of the upstream sensors, such as transmembrane osmosensor (*SHO1*) or histidine kinase osmosensor (*SLN1*), in the same signaling system are also partially tolerant to CW. Therefore, in addition to the cell wall integrity pathway, fungal susceptibility to cell wall interfering agents is also dependent upon the existence/availability of intact components of the antioxidant MAPK signaling pathways [6,7,8]. A similar drug tolerance was also determined in the human yeast pathogen *Candida albicans* [9]. Cross talks between MAPK routes, i.e., the “antioxidant” and “cell wall integrity” pathways, have recently been reported [10,11].

Antifungal chemosensitization is an intervention strategy for effective control of fungal pathogens, where co-administration of a chemosensitizer (viz., natural or synthetic compounds that function as intervention catalysts) increases the efficacy of the conventional drug co-administered [12]. A chemosensitizer on its own does not have to possess potent antifungal activity. However, sub-fungicidal concentration of a chemosensitizer significantly incapacitates fungal defense systems to a conventional drug, thus making the pathogen highly susceptible to the drug co-applied [12]. Examples of antifungal chemosensitization include: (1) piperazinyl quinolone, which sensitizes the human pathogen *C. albicans* to the azole drug fluconazole (FLC), resulting in overcoming FLC resistance of *C. albicans* [13], (2) cyclobutene-dione (squarile) derivatives, which sensitize the *C. albicans* major facilitator superfamily transporter (Mdr1p; responsible for FLC resistance) to FLC [14], and (3) benzhydroxamic acid (inhibitor of mitochondrial alternative oxidases), which enhances the sensitivity of *Rhizopus oryzae* to triazoles (posaconazole, itraconazole) [15].

The cinnamic acid derivatives are generally recognized as safe (GRAS) reagent [16], and thus, are currently used as food additives. Of note, cinnamic acid derivatives, such as cinnamic acid amides, have also been developed as antifungal agents by targeting cell wall biosynthesis [17,18].

In this study, the effectiveness of thirty-three cinnamic acid derivatives as intervention catalysts was investigated. Results showed 4-chloro-α-methyl- and 4-methylcinnamic acids (Figure 1) possessed the highest chemosensitizing capability to augment the efficacy of cell wall-targeting antifungals (CAS, octyl gallate (OG)) or to overcome fungal tolerance to the fungicide fludioxonil. It is concluded that cinnamic acid derivatives could be developed as promising intervention catalysts for effective control of fungal pathogens.

## 2. Results and Discussion

### 2.1. Identification of the Most Potent Cinnamic Acids via Yeast Screening: 4-Chloro-α-methyl-, 4-Methoxy-, 4-Methyl- and 3-Methylcinnamic Acids

To investigate the cell wall-interfering potential of cinnamic acids in fungi, the antifungal efficacy of 33 analogs of cinnamic acid (0.5 mM cutoff; Table 1) was investigated by examining cell wall integrity mutants of *S. cerevisiae* (*slt2*Δ and *bck1*Δ, encoding MAPK and MAPK kinase kinase (MAPKKK), respectively) in in vitro yeast dilution bioassays. The *S. cerevisiae bck1*Δ and *slt2*Δ have been serving as screening tools for identifying new cell wall disrupting agents, considering these mutants were highly susceptible to cell wall perturbing reagents such as CAS [19]. In the yeast pathogen *C. albicans*, CAS treatment also triggers increased expression of *GLR1*, which encodes the enzyme glutathione reductase responsible for maintaining cellular glutathione homeostasis [20]. Therefore, glutathione reductase mutant (*glr1*Δ) was also included in this study.

Initially, 4-chloro-α-methyl-, 4-methoxy-, 4-methyl- and 3-methylcinnamic acids were found to possess the highest antifungal activity against *S. cerevisiae* cell wall integrity mutants (viz., no growth of *bck1*Δ and *slt2*Δ at 0.5 mM) (Table 1). Test compounds were classified into three groups based on the level of antifungal activity against *bck1*Δ and *slt2*Δ mutants (at 0.5 mM) as follows: Group 1 (Four cinnamic acids), 4-chloro-α-methyl-, 4-methoxy-, 4-methyl-, 3-methylcinnamic acids (Complete growth inhibition of *bck1*Δ and *slt2*Δ (Growth score = 0)). The group 1 compounds also inhibited the growth of WT (Growth score = 0 to 1); Group 2 (Eight cinnamic acids), 2-chloro-α-methyl-, α-methyl-, 3,4-dimethoxy-, 4-ethoxy-3-methoxy-, benzyl-, 3,4-dihydroxy-*trans*-, 3-chloro-4-methoxy-, β-methylcinnamic acids (Moderate growth inhibition of *bck1*Δ and *slt2*Δ (Growth score = 2 to 5)); Group 3, the remaining twenty-two cinnamic acids (including cinnamic acid as the basic structure) (No growth inhibition of *bck1*Δ and *slt2*Δ (Growth score = 6)) (Table 1).

**Figure 2:**
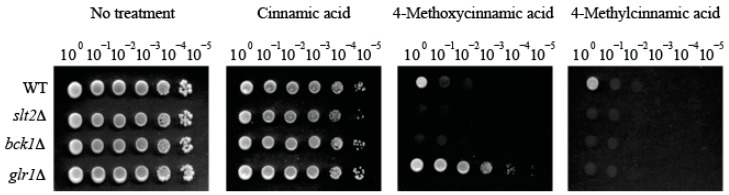
Yeast dilution bioassay showing differential susceptibility of *S. cerevisiae slt2*Δ, *bck1*Δ, and *glr1*Δ mutants to cinnamic acid analogs (0.5 mM).

Notably, the *glr1*Δ mutant was hyper-tolerant (Growth score = 6) to 4-methoxycinnamic acid when compared to other strains (WT, *bck1*Δ, *slt2*Δ; Growth score = 0 to 1). However, this hyper-tolerance was abolished by 4-methylcinnamic acid (Growth score = 0), the deoxygenated analog of 4-methoxycinnamic acid (at *para* methoxyl moiety; Table 1; Figure 2). In contrast, treatment with the other deoxygenated analog 3-methylcinnamic acid (Group 1) could not abolish hyper-tolerance of *glr1*Δ mutant (Growth score = 6; Table 1; Figure data not shown), indicating structure-activity relationship of test compounds, where *para* substitution of methyl moiety is crucial for overcoming the *glr1*Δ hyper-tolerance to 4-methoxycinnamic acid. 

### 2.2. Fungal Tolerance to 4-Methoxycinnamic Acid Was Unique to Glutathione Reductase Mutant

*GLR1* encodes the enzyme glutathione reductase (cytosolic and mitochondrial), which converts oxidized glutathione (GSSG) to reduced glutathione (GSH) [21] (Supplementary Figure S1). Cytosolic glutathione reductase also determines the redox state of glutathione in the mitochondrial inter-membrane space [22]. *GLR1* is known to genetically interact with thirteen genes involved in: (1) Cell redox homeostasis, (2) Cellular response to oxidative stress, (3) Protein glutathionylation, and (4) Glutathione metabolic process (Table 2; Supplementary Figure S2) [22]. Therefore, another yeast bioassay was performed to investigate whether *GLR1* interacting gene mutants were also hyper-tolerant to 4-methoxycinnamic acid. 

In a prior study, the cell wall disrupting drug CAS also activated fungal antioxidant system, viz., enhanced expression of Mn-superoxide dismutase (*SOD2*) and nuclear relocation of MAPK (Hog1p) that leads to incremental increase of catalase (*CAT1*) expression/activity [20]. Thus, four additional mutants of antioxidant genes—two catalases (catalases A, T) and two superoxide dimutases (cytosolic, mitochondrial) mutants—were also included in this assay (Table 2).

As shown in Table 2, hyper-tolerant response to 4-methoxycinnamic acid was unique to *glr1*Δ mutant. All other mutants (including WT) were hyper-sensitive to the same treatment (Growth score = 0 to 1). Therefore, results indicated that functions of “*GLR1*-interacting” or “CAS-responsive” antioxidant genes were not correlated with *glr1*Δ hyper-tolerance to 4-methoxycinnamic acid. 

### 2.3. Supplementation of Reduced Glutathione (GSH) Did Not Abolish Hyper-Tolerance to 4-Methoxycinnamic Acid 

**Figure 3:**
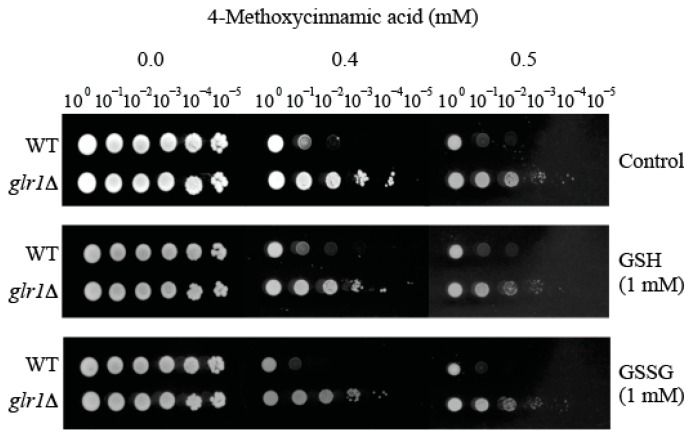
Glutathione supplementation test. The tolerance of *S. cerevisiae* glutathione reductase mutant (*glr1*Δ) to 4-methoxycinnamic acid was not abolished by supplementation of reduced (GSH) or oxidized (GSSG) glutathione, indicating glutathione limitation was not the determinant of *glr1*Δ hyper-tolerance to 4-methoxycinnamic acid.

To determine whether supplementation of GSH (Reduced glutathione; the metabolic product of glutathione reductase) can abolish the hyper-tolerance of *glr1*Δ mutant to 4-methoxycinnamic acid, yeast bioassay was performed by exogenously providing GSH (1 mM) into yeast bioassay medium (See Figure 3). For comparison, the effect of GSSG (Oxidized glutathione; 1 mM) was also investigated. As shown in Figure 3, *glr1*Δ hyper-tolerance to 4-methoxycinnamic acid was not abolished by GSH (or GSSG), thus indicating limitation of cellular GSH caused by *GLR1* mutation was not the determinant of *glr1*Δ hyper-tolerance to 4-methoxycinnamic acid. 

Currently, it is not well understood how the methoxyl- or methyl- moiety in cinnamic acid at the *para* substitution is correlated with hyper-tolerance or hyper-sensitivity, respectively, of *glr1*Δ mutant. 4-Methoxycinnamic acid is a natural phenolic acid, which is previously shown to possess therapeutic potential such as prevention of colon carcinogenesis, increase in insulin secretion, etc. [23,24]. Whereas, except for few reports on metabolism of 4-methylcinnamic acid by certain *Clostridium* spp. [25], studies on bioactivity of 4-methylcinnamic acid are very limited. 

Noteworthy is that methyl-replaced anti-tuberculosis drug possessed higher antibacterial activity when compared to the methoxyl-containing drug [26]. For instance, moxifloxacin (containing “8-methoxyl” moiety) is one of the fluoroquinolones for treating tuberculosis, especially multidrug-resistant infections [26]. Moxifloxacin interacts with the DNA gyrase, the DNA strand-breaking enzyme [26]. Certain mutations in DNA gyrase trigger bacterial drug resistance, which is due to the disruption of bridge—enzyme interaction (thus, decreasing drug affinity). However, introduction of “8-methyl” moiety into moxifloxacin made the modified drug more potent compared to the 8-methoxy-moxifloxacin against gyrase, especially the mutant enzyme causing drug resistance [26]. 

Therefore, it is surmised that, as observed in moxifloxacin, 4-methylcinnamic acid could also possess higher affinity to the cellular target(s) when compared to 4-methoxycinnamic acid, resulting in better control of fungal pathogens. Precise determination of the mechanism as to overcoming *glr1*Δ hyper-tolerance (to 4-methoxycinnamic acid) by 4-methylcinnamic acid warrants future study. 

### 2.4. Overcoming Fludioxonil Tolerance of Aspergillus Fumigatus Antioxidant MAPK Mutants by 4-Chloro-α-methylcinnamic Acid

Fludioxonil is a commercial phenylpyrrole fungicide, which triggers abnormal and excessive stimulation of the “antioxidant” MAPK signaling system [27]. This abnormal activation of antioxidant MAPK pathway results in cellular energy deprivation via metabolic shifts from normal fungal growth to exhaustive oxidative stress response. Accordingly, application of fludioxonil prevents the growth of fungal pathogens. However, as observed in cell wall–disrupting drugs or agents (see Introduction), fungi having mutations in components of upstream signaling system, viz., antioxidant MAPK signaling pathway, can escape fludioxonil (10 to 100 μg/mL) toxicity [27]. 

**Figure 4:**
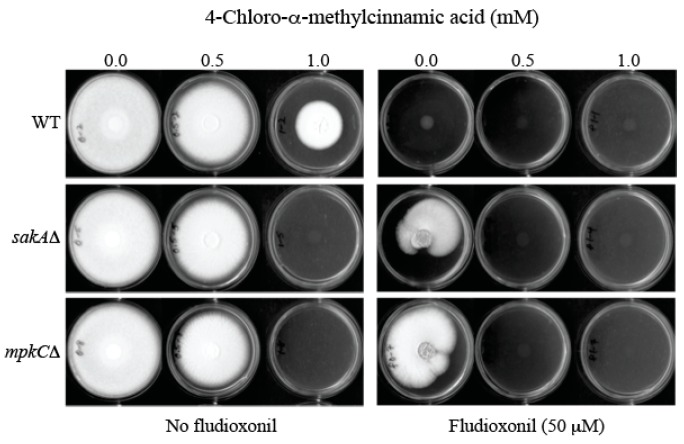
Overcoming fludioxonil (50 μM) tolerance of *A. fumigatus* mitogen-activated protein kinase (MAPK) mutants (*sakA*Δ, *mpkC*Δ) by 4-chloro-α-methylcinnamic acid (0.5 mM).

The *sakA*Δ and *mpkC*Δ are antioxidant MAPK mutants of the human pathogen *A. fumigatus* [28,29]. As shown in Figure 4, *A. fumigatus* *sakA*Δ and *mpkC*Δ mutants exhibited tolerance to fludioxonil (50 μM, equivalent to 12.5 μg/mL), thus developing radial growth (*sakA*Δ: 66.7 ± 20.0%; *mpkC*Δ: 72.3 ± 31.0%; Relative growth compared to “no treatment” control (100.0 ± 0.0%)) on potato dextrose agar (PDA), whereas the growth of WT was completely inhibited. However, co-application of sub-fungicidal concentration of 4-chloro-α-methylcinnamic acid (0.5 mM) with fludioxonil (50 μM) effectively prevented fungal tolerance to fludioxonil, thus achieving complete growth inhibition of MAPK mutants (Figure 4). (Note: 4-Methyl- or 4-methoxycinnamic acid did not overcome fludioxonil tolerance of *A. fumigatus* MAPK mutants at the same test conditions—data not shown.) 

Of note, *A. fumigatus* MAPK mutants exhibited higher sensitivity to the independent treatment of 4-chloro-α-methylcinnamic acid, alone, when compared to the WT (Figure 4). For example, *A. fumigatus* WT developed radial growth on PDA at 1.0 mM of 4-chloro-α-methylcinnamic acid, while the growth of MAPK mutants (*sakA*Δ, *mpkC*Δ) was completely inhibited at the same condition. 

Natural phenolic agents or their structural analogs are potent redox cyclers in cells, thus inhibiting fungal growth via disruption of cellular redox homeostasis or redox-sensitive cellular components [30,31]. Therefore, it is speculated that 4-chloro-α-methylcinnamic acid (as a redox cycler) further targets fungal antioxidant system, such as MAPK pathway. Accordingly, in addition to interfering with cell wall integrity, debilitation of cellular antioxidant system could also be one mechanism of antifungal action of 4-chloro-α-methylcinnamic acid. Fungi with mutated MAPK systems (e.g., *A. fumigatus* *sakA*Δ, *mpkC*Δ) are incapable of initiating a fully operational defense against oxidative stress triggered by 4-chloro-α-methylcinnamic acid, thus resulting in increased growth inhibition.

Collectively, 4-chloro-α-methylcinnamic acid (0.5 mM) could function as an intervention catalyst to overcome fludioxonil (50 μM) tolerance of fungal pathogens. 

### 2.5. Antifungal Chemosensitization of 4-Chloro-α-methyl- or 4-Methylcinnamic Acid to CAS or OG

Antifungal chemosensitization to enhance antifungal potency of CAS was investigated in filamentous fungus *Aspergillus brasiliensis* ATCC16404 according to the methods outlined by the Clinical Laboratory Standards Institute (CLSI) M38-A [32]. 4-Chloro-α-methyl- and 4-methylcinnamic acids (possessing the highest antifungal efficacy) were examined for their potential as chemosensitizers (i.e., intervention catalysts) (4-Methoxycinnamic acid was also included in the test for comparison). Both minimum inhibitory concentrations (MICs) and minimum fungicidal concentrations (MFCs), and thus, both fractional inhibitory concentration indices (FICIs) and fractional fungicidal concentration indices (FFCIs) of fungus were determined (See Experimental section for calculation). *A. brasiliensis* is one of the test microorganisms (mold) for evaluating antifungal efficacy of industrial products [33]. CAS, like other echinocandin drugs, generally cannot achieve complete inhibition of the growth of filamentous fungal pathogens [34], thus resulting in fungal survival during CAS treatment.

OG, an alkyl derivative of the natural product gallic acid, was also included in this chemosensitization test as a reference phenolic compound. OG is classified as a GRAS food additive [16], and is recently determined as a safer, more effective preservative for consumer products [35]. OG was previously shown to negatively affect the growth of cell wall MAPK mutants [36]. 

For FICIs of 4-chloro-α-methylcinnamic acid, despite the absence of calculated synergism, there was increased antifungal activity of 4-chloro-α-methylcinnamic acid and CAS or OG (viz., chemosensitizing effect; FICIs = 1.0), which was reflected in lowered MICs of 4-chloro-α-methylcinnamic acid and CAS or OG when compounds were co-applied (Table 3). For instance, co-application of 4-chloro-α-methylcinnamic acid (1.6 mM) with CAS (16 μg/mL) or OG (0.05 mM) completely inhibited the growth of *A. brasiliensis*, while individual treatment of each compound, alone, at the same concentrations allowed the survival of fungus (Table 3).

Similar trends were also observed in FICIs of 4-methylcinnamic acid. There was increased antifungal activity of 4-methylcinnamic acid and CAS or OG (viz., chemosensitizing effect; FICIs = 0.8 to 1.0) in *A. brasiliensis*, which was reflected in lowered MICs of 4-methylcinnamic acid and CAS or OG when compounds were co-applied. For example, co-application of 4-methylcinnamic acid (6.4 mM) with CAS (8 μg/mL) or OG (0.05 mM) completely inhibited the growth of *A. brasiliensis*, while individual treatment of each compound, alone, at the same concentrations allowed the growth of fungus (Table 3). In comparison, no chemosensitizing capability was found with 4-methoxycinnamic acid (FICIs = 2.0; Table 3).

For FFCIs, no chemosensitizing capability was determined in any of the combinations of compounds tested, indicating chemosensitizing capability of 4-chloro-α-methyl- or 4-methylcinnamic acid was at the level of lowering MICs (but not MFCs) of test compounds (Table 3). 

All together, results suggest that 4-chloro-α-methyl- or 4-methylcinnamic acid could be developed as potent intervention catalysts/chemosensitizers for improved fungal control.

Antifungal activities of various cinnamic acid derivatives have been documented elsewhere [37,38,39]. Structure-activity relationship revealed that the substitution design of cinnamic phenyl ring significantly affected antifungal activity [39]. For instance, introducing substituents to the ring structure of “ethyl” cinnamic acid derivatives generally resulted in enhancement of antifungal activity as follows: (1) w/electron-donating groups (hydroxyl, methoxyl): Order of antifungal activity was *o*-substitution > *p*-substitution > *m*-substitution, (2) w/electron-withdrawing groups (halogen atoms, such as chlorine): Highest activity was observed with *p*-substitution [39].

With regard to electron-donating groups, our results from cinnamic acid analogs were not well aligned with that of “ethyl” cinnamic acid derivatives [39]. For example, in our study, 4-methoxycinnamic acid (*p*-substitution) belongs to the Group 1, possessing the highest antifungal activity (Table 1), while 2- and 3-methoxycinnamic acids (*o*- and *m*-substitution, respectively) belong to Group 3, showing no antifungal activity (Table 1). 

Whereas for electron-withdrawing group, like the “ethyl” cinnamic acid [39], enhanced antifungal activity with *para* substitution was also observed in our study. For example, while 2-chloro-α-methylcinnamic acid (*o*-chlorine) exhibited only moderate antifungal activity (Group 2; Table 1), its analog 4-chloro-α-methylcinnamic acid (*p*-chlorine) possessed the highest antifungal potency (Group 1; Table 1). Thus, our results with chlorine-containing cinnamic acid were well aligned with that of “ethyl” cinnamic acid derivatives [39]. Results further indicated that basic structure of cinnamic acids, e.g., cinnamic acid vs. “ethyl” cinnamic acid (as compared above), also affects differential antifungal activity when substituents are introduced/added to the phenyl ring. Elucidation of comprehensive structure-activity relationship of cinnamic acid derivatives warrants future in-depth investigation.

**Figure 5:**
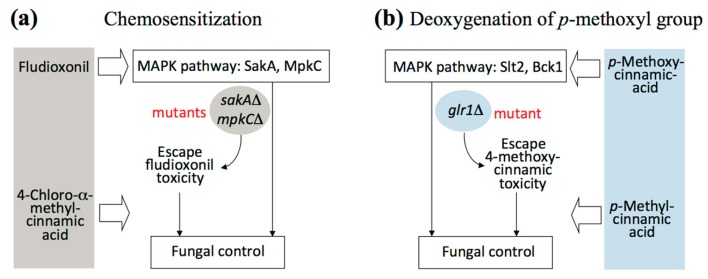
Scheme showing overcoming fungal tolerance to antifungal agents. (**a**) Chemosensitization to overcome fludioxonil tolerance of *A. fumigatus* MAPK mutants by small molecule chemosensitizer 4-chloro-α-methylcinnamic acid; (**b**) Deoxygenation of 4-methoxyl group to overcome 4-methoxycinnamic acid tolerance of *S. cerevisiae glr1*Δ mutant.

In summary, use of small molecules as intervention catalysts could serve as an alternative strategy for effective control of fungal pathogens. Improvement of the efficacy of CAS or OG was achieved by 4-chloro-α-methyl- or 4-methylcinnamic acid-mediated chemosensitization. 4-Chloro-α-methylcinnamic acid further overcame fludioxonil tolerance of *A. fumigatus* antioxidant MAPK mutants when co-applied (See Figure 5a for scheme). Modification of chemical structures, such as deoxygenation of *para* substituted methoxyl moiety of cinnamic acid (See Figure 5b for scheme), could also abolish *glr1*Δ tolerance to 4-methoxycinnamic acid. 

## 3. Materials and Methods 

### 3.1. Fungal Strains and Culture Conditions 

*Aspergillus fumigatus* AF293 wild type (WT), *A. fumigatus* MAPK gene deletion mutants (*sakA*Δ, *mpkC*Δ) [28,29] and *Aspergillus brasiliensis* ATCC16404 were cultured at 35 °C or 28 °C, respectively, on potato dextrose agar (PDA). *Saccharomyces cerevisiae* BY4741 wild type (*Mat* a *his3*Δ*1 leu2*Δ*0* *met15*Δ*0* *ura3*Δ*0*) and twenty-one selected single gene deletion mutants (Cell wall integrity system mutants: *bck1*Δ, *slt2*Δ; Glutathione (GSH) homeostasis mutants: *glr1*Δ, *gsh1*Δ, *gsh2*Δ; Twelve *GLR1* interacting gene mutants (Table 2); Four caspofungin (CAS) responsive antioxidant gene mutants (Table 2)) were procured from Invitrogen (Carlsbad, CA, USA) and Open Biosystems (Huntsville, AL, USA; See also [22]). Yeasts were cultured on Synthetic Glucose (SG; Yeast nitrogen base without amino acids 0.67%, glucose 2% with appropriate supplements: Uracil 0.02 mg/mL, amino acids 0.03 mg/mL) or Yeast Peptone Dextrose (YPD; Bacto yeast extract 1%, Bacto peptone 2%, glucose 2%) medium at 30 °C. All chemicals for culturing fungi or yeast were procured from Sigma Co. (St. Louis, MO, USA).

### 3.2. Antifungal Susceptibility Testing

#### 3.2.1. Yeast Dilution Bioassay in *Saccharomyces cerevisiae*

Petri plate-based yeast dilution bioassays were performed on the WT and gene deletion mutants to determine their level of susceptibility to thirty-four cinnamic acid derivatives (Test concentrations: 0.1, 0.2, 0.3, 0.4, 0.5, and 1.0 mM; Table 1). 1 × 10^6^ cells of the WT or mutants of *S. cerevisiae*, cultured on YPD plates, were serially diluted 10-fold in SG liquid medium supplemented with amino acids and uracil (See above) five times to yield cell dilution cohorts of 10^6^, 10^5^, 10^4^, 10^3^, 10^2^, and 10^1^ cells. Cells from each dilution of respective strains were spotted on SG agar incorporated with individual compounds, such as cinnamic acid analogs. Yeast cells were incubated at 30 °C (5 to 7 days). Results were monitored/evaluated based on a designated (log10) value of the highest dilution where colonies became visible (Score “0”—no colonies were visible from any of the dilution spots, Score “1”—only colonies from the spot with 10^6^ cells, Score “2” only colonies from the spots with 10^6^ and 10^5^ cells were visible, etc., while Score “6”—colonies were visible from all dilution spots). Therefore, each unit (1 to 6) of numerical difference was equivalent to a 10-fold difference in the sensitivity of test strain to the treatment.

Yeast dilution bioassays were also performed on the WT and *glr1*Δ, as described above, to assess effects of reduced or oxidized glutathione (GSH or GSSG, respectively; 1 mM) on reverting 4-methoxycinnamic acid tolerance of *glr1*Δ.

Compounds were dissolved in dimethylsulfoxide (DMSO; absolute DMSO amount: <2% in media) before incorporation into culture media. Throughout this study, control plates (No treatment) contained DMSO at levels equivalent to that of cohorts receiving antifungal agents, within the same set of experiments.

#### 3.2.2. Agar Plate Bioassay in *Aspergillus fumigatus*: Susceptibility of WT and MAPK Mutants to Cinnamic Acid Derivatives

Determination of overcoming fludioxonil tolerance of *A. fumigatus sakA*Δ and *mpkC*Δ mutants was based on comparison of fungal radial growth between treated and control colonies (See Figure 4). Fungal conidia (5 × 10^3^) were diluted in phosphate buffered saline and inoculated as a drop onto the center of PDA plates containing: (1) No treatment (control); (2) 4-chloro-α-methyl-, 4-methyl-, or 4-methoxycinnamic acid (0.1, 0.2, 0.3, 0.4, 0.5, 0.6, 0.7, 0.8, 0.9, 1.0 mM); (3) fludioxonil (50 μM); and (4) 4-chloro-α-methyl-, 4-methyl-, or 4-methoxycinnamic acid + fludioxonil. Growth was observed for five to seven days at 35 °C. 

#### 3.2.3. Liquid Bioassay in *Aspergillus brasiliensis* (CLSI Protocol)

To determine the precise level of compound interaction between cinnamic acid derivatives (0.1, 0.2, 0.4, 0.8, 1.6, 3.2, 6.4 mM) and other antifungal agents (Octyl gallate (OG) (0.00625, 0.0125, 0.025, 0.05, 0.1, 0.2, 0.4, 0.8, 1.6 mM); CAS (0.0625, 0.125, 0.25, 0.5, 1.0, 2.0, 4.0, 8.0, 16.0 μg/mL)) in the filamentous fungus *Aspergillus brasiliensis* ATCC16404, checkerboard bioassays (triplicate) (0.4 × 10^4^–5 × 10^4^ CFU/mL) were performed in microtiter wells using a broth microdilution method (in RPMI 1640 medium; Sigma Co., St. Louis, MO, USA), according to the protocol outlined by the Clinical and Laboratory Standards Institute (CLSI) M38-A [32]. RPMI 1640 medium was supplemented with 0.03% L-glutamine and buffered with 0.165 mM 3-(*N*-morpholino) propanesulfonic acid (Sigma Co., St. Louis, MO, USA). Minimum inhibitory concentrations (MICs), lowest concentration of agents showing no visible fungal growth in microtiter wells (200 μL per well), were assessed after 48 h. Minimum fungicidal concentrations (MFCs), lowest concentration of agents achieving ≥99.9% fungal death, were determined following completion of MIC assays by spreading entire volumes of microtiter wells (200 μL) onto individual PDA (recovery) plates, and culturing for additional 48 h (at 28 °C). Compound interactions, i.e., Fractional Inhibitory Concentration Indices (FICIs) and Fractional Fungicidal Concentration Indices (FFCI), were calculated as follows: FICI or FFCI = (MIC or MFC of compound A in combination with compound B/MIC or MFC of compound A, alone) + (MIC or MFC of compound B in combination with compound A/MIC or MFC of compound B, alone). Levels and types of compound interactions between antifungal agents (Cinnamic acid derivatives and CAS or OG) were defined as: synergistic (FICI or FFCI ≤ 0.5) or indifferent (FICI or FFCI > 0.5–4) [40]. 

### 3.3. Statistical Analysis

Statistical analysis (student’s *t*-test) was performed based on “Statistics to use” [41], where *p* < 0.05 was considered significant.

## 4. Conclusions

Antifungal potency of 33 cinnamic acid derivatives was investigated. The efficacy of CAS or OG, the cell wall disrupting agents, was augmented by 4-chloro-α-methyl- or 4-methylcinnamic acid screened. Antifungal chemosensitization by 4-chloro-α-methyl- or 4-methylcinnamic acid greatly lowers MICs of antifungal agents co-administered. 4-Chloro-α-methylcinnamic acid further overcame fludioxonil tolerance of *A. fumigatus* antioxidant MAPK mutants. 4-Methylcinnamic acid, the deoxygenated analog of 4-methoxycinnamic acid, also overcame *glr1*Δ tolerance to 4-methoxycinnamic acid, indicating the importance of *para* substitution of methyl moiety for improved antifungal activity. Collectively, 4-chloro-α-methyl- and 4-methylcinnamic acids possess chemosensitizing capability to enhance the efficacy of CAS, OG, or fludioxonil and thus can be developed as target-based (namely, cell wall disruption) intervention catalysts.

## Figures and Tables

**Table 1:** Growth scores of yeast strains at 0.5 mM (cutoff) of cinnamic acid analogs during yeast dilution bioassay (WT, Wild type; 0, No growth; 6, Full growth; See Experimental section).

Cinnamic Acids	WT	*slt2*Δ	*bck1*Δ	*glr1*Δ
Group 1 (Highest activity): 4-Chloro-α-methylcinnamic acid	0	0	0	0
4-Methylcinnamic acid	1	0	0	0
4-Methoxycinnamic acid	1	0	0	6
3-Methylcinnamic acid	1	0	0	6
Group 2 (Moderate activity): 2-Chloro-α-methylcinnamic acid	6	2	2	4
α-Methylcinnamic acid	6	4	6	6
3,4-Dimethoxycinnamic acid	6	4	6	6
4-Ethoxy-3-methoxycinnamic acid	6	4	6	6
Benzylcinnamic acid	6	5	5	6
3,4-Dihydroxy-*trans*-cinnamic acid	6	5	5	6
3-Chloro-4-methoxycinnamic acid	6	5	6	6
β-Methylcinnamic acid	6	5	6	6
Group 3 (No activity): Cinnamic acid (Basic structure)	6	6	6	6
Methyl-*trans*-cinnamic acid	6	6	6	6
2-Methylcinnamic acid	6	6	6	6
2-Methoxycinnamic acid	6	6	6	6
3-Methoxycinnamic acid	6	6	6	6
3,4,5-Trimethoxycinnamic acid	6	6	6	6
4-Hydroxy-3-methoxycinnamic acid	6	6	6	6
3-Hydroxy-4-methoxycinnamic acid	6	6	6	6
2-Hydroxycinnamic acid	6	6	6	6
3-Hydroxycinnamic acid	6	6	6	6
4-Hydroxycinnamic acid	6	6	6	6
Cinnamyl acetate	6	6	6	6
4-Acetoxy-3-methoxycinnamic acid	6	6	6	6
4-Hexadecyloxy-3-methoxycinnamic acid	6	6	6	6
2-Methoxy-α-methylcinnamic acid	6	6	6	6
Methyl α-methylcinnamic acid	6	6	6	6
Ethyl 4-hydroxy-3-methoxycinnamic acid	6	6	6	6
4-Benzyloxy-3-methoxycinnamic acid	6	6	6	6
3,5-Dimethoxy-4-hydroxycinnamic acid	6	6	6	6
4-Amino-2-methylcinnamic acid	6	6	6	6
3,4-Dihydroxyhydrocinnamic acid	6	6	6	6
3-Hydroxy-α-mercapto-β-methylcinnamic acid	6	6	6	6

**Table 2:** Responses of *S. cerevisiae* WT and gene deletion mutants to 4-methoxy- or 4-methylcinnamic acid (0.5 mM).

*S. cerevisiae*	Functions of Deleted Genes	4-Methoxy-	4-Methyl-
WT	Parental (*Mat* a *his3*Δ*1 leu2*Δ*0 met15*Δ*0 ura3*Δ*0*)	1	1
*glr1*Δ	*GLR1* & *GLR1* interacting genes: Glutathione reductase	4 (Hyper-tolerant)	0
*trr1*Δ	Cytoplasmic thioredoxin reductase	0	0
*trr2*Δ	Mitochondrial thioredoxin reductase	1	0
*tsa1*Δ	Thioredoxin peroxidase	0	0
*trx1*Δ	Cytoplasmic thioredoxin isoenzyme	0	0
*trx2*Δ	Cytoplasmic thioredoxin isoenzyme	0	0
*gsh1*Δ	γ-glutamylcysteine synthetase	0	0
*gsh2*Δ	Glutathione synthetase	0	0
*grx1*Δ	Glutathione-dependent disulfide oxidoreductase	1	0
*grx2*Δ	Cytoplasmic glutaredoxin	0	0
*ahp1*Δ	Thiol-specific peroxiredoxin	1	0
*prx1*Δ	Mitochondrial peroxiredoxin	0	0
*tsa2*Δ	Cytoplasmic thioredoxin peroxidase	0	0
*dot5*Δ	Nuclear thiol peroxidase	0	0
*ycf1*Δ	Vacuolar glutathione *S*-conjugate transporter	1	0
*ctt1*Δ	CAS responsive antioxidant genes: Cytosolic catalase T	0	0
*cta1*Δ	Catalase A	0	0
*sod1*Δ	Cytosolic Cu,Zn superoxide dismutase	0	0
*sod2*Δ	Mitochondrial Mn superoxide dismutase	0	0

**Table 3:** Antifungal chemosensitization of 4-chloro-α-methyl- and 4-methylcinnamic acids to caspofungin (CAS) (μg/mL) or OG (mM), tested against *Aspergillus* *brasiliensis*: Summary of Clinical Laboratory Standards Institute (CLSI) bioassays.

Compounds	MIC Alone	MIC Combined	FICI	MFC Alone	MFC Combined	FFCI
4-Chloro-α-methyl	3.2	1.6	1	12.8	12.8	2
CAS	32	16		32	32	
4-Methyl	12.8	6.4	0.8	12.8	12.8	2
CAS	32	8		32	32	
4-Methoxy	12.8	12.8	2	12.8	12.8	2
CAS	32	32		32	32	
Mean: Chemosensitizers	9.6	6.9	−	12.8	12.8	−
CAS	32	18.7	−	32	32	−
*t*-test ^1^: Chemosensitizers	−	*p* = 0.590	−	−	*p* = 1.000	−
CAS	−	*p* = 0.132	−	−	*p* = 1.000	−
4-Chloro-α-methyl	3.2	1.6	1	12.8	12.8	2
OG	0.1	0.05		0.4	0.4	
4-Methyl	12.8	6.4	1	12.8	12.8	2
OG	0.1	0.05		0.4	0.4	
4-Methoxy	12.8	12.8	2	12.8	12.8	2
OG	0.1	0.1		0.4	0.4	
Mean: Chemosensitizers	9.6	6.9	−	12.8	12.8	−
OG	0.1	0.07	−	0.4	0.4	−
*t*-test ^1^: Chemosensitizers	−	*p* = 0.590	−	−	*p* = 1.000	−
OG	−	*p* = 0.116	−	−	*p* = 1.000	−

^1^ Student’s *t*-test for paired data (combined, i.e., chemosensitization) was vs. mean minimum inhibitory concentration (MIC) or minimum fungicidal concentrations (MFC) of each compound (alone, i.e., no chemosensitization) determined in strains. Note that *p* values for chemosensitization were determined as >0.05.

## Supplementary Materials

The following are available online, Supplementary Figure S1. Diagram showing the role of glutathione reductase to reduce GSSG (Oxidized glutathione) to GSH (Reduced glutathione) (Adapted from [21]), Supplementary Figure S2. Diagram showing *GLR1* interacting genes. Functions of *GLR1* interacting genes include: (1) Cell redox homeostasis, (2) Cellular response to oxidative stress, (3) Protein glutathionylation, and (4) Glutathione metabolic process (Downloaded from *Saccharomyces cerevisiae* Genome Database; [22]).

## References

[1] Latge J.P., Beauvais A., Chamilos G. (2017). The cell wall of the human fungal pathogen *Aspergillus fumigatus*: Biosynthesis, organization, immune response, and virulence. Annu. Rev. Microbiol..

[2] Perlin D.S. (2015). Mechanisms of echinocandin antifungal drug resistance. Ann. N. Y. Acad. Sci..

[3] Levin D.E., Fields F.O., Kunisawa R., Bishop J.M., Thorner J. (1990). A candidate protein kinase C gene, *PKC1*, is required for the *S. cerevisiae* cell cycle. Cell.

[4] Fuchs B.B., Mylonakis E. (2009). Our paths might cross: The role of the fungal cell wall integrity pathway in stress response and cross talk with other stress response pathways. Eukaryot. Cell.

[5] Fujioka T., Mizutani O., Furukawa K., Sato N., Yoshimi A., Yamagata Y., Nakajima T., Abe K. (2007). MpkA-dependent and -independent cell wall integrity signaling in *Aspergillus nidulans*. Eukaryot. Cell.

[6] García-Rodriguez L.J., Durán A., Roncero C. (2000). Calcofluor antifungal action depends on chitin and a functional high-osmolarity glycerol response (HOG) pathway: Evidence for a physiological role of the *Saccharomyces cerevisiae* HOG pathway under noninducing conditions. J. Bacteriol..

[7] Jiang B., Ram A.F., Sheraton J., Klis F.M., Bussey H. (1995). Regulation of cell wall beta-glucan assembly: *PTC1* negatively affects *PBS2* action in a pathway that includes modulation of *EXG1* transcription. Mol. Gen. Genet..

[8] Lai M.H., Silverman S.J., Gaughran J.P., Kirsch D.R. (1997). Multiple copies of *PBS2*, *MHP1* or *LRE1* produce glucanase resistance and other cell wall effects in *Saccharomyces cerevisiae*. Yeast.

[9] Alonso-Monge R., Navarro-García F., Molero G., Diez-Orejas R., Gustin M., Pla J., Sánchez M., Nombela C. (1999). Role of the mitogen-activated protein kinase Hog1p in morphogenesis and virulence of *Candida albicans*. J. Bacteriol..

[10] Altwasser R., Baldin C., Weber J., Guthke R., Kniemeyer O., Brakhage A.A., Linde J., Valiante V. (2015). Network modeling reveals cross talk of MAP kinases during adaptation to caspofungin stress in *Aspergillus fumigatus*. PLoS ONE.

[11] Rodríguez-Peña J.M., García R., Nombela C., Arroyo J. (2010). The high-osmolarity glycerol (HOG) and cell wall integrity (CWI) signalling pathways interplay: A yeast dialogue between MAPK routes. Yeast.

[12] Campbell B.C., Chan K.L., Kim J.H. (2012). Chemosensitization as a means to augment commercial antifungal agents. Front. Microbiol..

[13] Youngsaye W., Hartland C.L., Morgan B.J., Ting A., Nag P.P., Vincent B., Mosher C.A., Bittker J.A., Dandapani S., Palmer M. (2013). ML212: A small-molecule probe for investigating fluconazole resistance mechanisms in *Candida albicans*. Beilstein J. Org. Chem..

[14] Keniya M.V., Fleischer E., Klinger A., Cannon R.D., Monk B.C. (2015). Inhibitors of the *Candida albicans* major facilitator superfamily transporter Mdr1p responsible for fluconazole resistance. PLoS ONE.

[15] Shirazi F., Kontoyiannis D.P. (2013). Mitochondrial respiratory pathways inhibition in *Rhizopus oryzae* potentiates activity of posaconazole and itraconazole via apoptosis. PLoS ONE.

[16] U.S. Food and Drug Administration (FDA) (2011). Everything Added to Food in the United States. http://www.fda.gov/Food/IngredientsPackagingLabeling/FoodAdditivesIngredients/ucm115326.htm.

[17] Fungicide Resistance Action Committee. http://www.frac.info.

[18] Ma C.M., Abe T., Komiyama T., Wang W., Hattori M., Daneshtalab M. (2010). Synthesis, anti-fungal and 1,3-beta-d-glucan synthase inhibitory activities of caffeic and quinic acid derivatives. Bioorg. Med. Chem..

[19] Reinoso-Martín C., Schüller C., Schuetzer-Muehlbauer M., Kuchler K. (2003). The yeast protein kinase C cell integrity pathway mediates tolerance to the antifungal drug caspofungin through activation of Slt2p mitogen-activated protein kinase signaling. Eukaryot. Cell.

[20] Kelly J., Rowan R., McCann M., Kavanagh K. (2009). Exposure to caspofungin activates Cap and Hog pathways in *Candida albicans*. Med. Mycol..

[21] Couto N., Wood J., Barber J. (2016). The role of glutathione reductase and related enzymes on cellular redox homoeostasis network. Free Radic. Biol. Med..

[22] *Saccharomyces* Genome Database. http://www.yeastgenome.org.

[23] Adisakwattana S., Hsu W.H., Yibchok-anun S. (2011). Mechanisms of p-methoxycinnamic acid-induced increase in insulin secretion. Horm. Metab. Res..

[24] Gunasekaran S., Venkatachalam K., Jeyavel K., Namasivayam N. (2014). Protective effect of *p*-methoxycinnamic acid, an active phenolic acid against 1,2-dimethylhydrazine-induced colon carcinogenesis: Modulating biotransforming bacterial enzymes and xenobiotic metabolizing enzymes. Mol. Cell Biochem..

[25] Chamkha M., Garcia J.L., Labat M. (2001). Metabolism of cinnamic acids by some Clostridiales and emendation of the descriptions of *Clostridium aerotolerans*, *Clostridium celerecrescens* and *Clostridium xylanolyticum*. Int. J. Syst. Evol. Microbiol..

[26] Aldred K.J., Blower T.R., Kerns R.J., Berger J.M., Osheroff N. (2016). Fluoroquinolone interactions with *Mycobacterium tuberculosis* gyrase: Enhancing drug activity against wild-type and resistant gyrase. Proc. Natl. Acad. Sci. USA.

[27] Kojima K., Takano Y., Yoshimi A., Tanaka C., Kikuchi T., Okuno T. (2004). Fungicide activity through activation of a fungal signalling pathway. Mol. Microbiol..

[28] Reyes G., Romans A., Nguyen C.K., May G.S. (2006). Novel mitogen-activated protein kinase MpkC of *Aspergillus fumigatus* is required for utilization of polyalcohol sugars. Eukaryot. Cell.

[29] Xue T., Nguyen C.K., Romans A., May G.S. (2004). A mitogen-activated protein kinase that senses nitrogen regulates conidial germination and growth in *Aspergillus fumigatus*. Eukaryot. Cell.

[30] Guillen F., Evans C.S. (1994). Anisaldehyde and veratraldehyde acting as redox cycling agents for H_2_O_2_ production by *Pleurotus eryngii*. Appl. Environ. Microbiol..

[31] Jacob C. (2006). A scent of therapy: Pharmacological implications of natural products containing redox-active sulfur atoms. Nat. Prod. Rep..

[32] Clinical and Laboratory Standards Institute (CLSI) (2008). Reference Method for Broth Dilution Antifungal Susceptibility Testing of Filamentous Fungi: Approved Standard–Second Edition. CLSI Document M38-A2.

[33] Moser C.L., Meyer B.K. (2011). Comparison of compendial antimicrobial effectiveness tests: A review. AAPS PharmSciTech.

[34] Walker L.A., Lee K.K., Munro C.A., Gow N.A. (2015). Caspofungin treatment of *Aspergillus fumigatus* results in ChsG-dependent upregulation of chitin synthesis and the formation of chitin-rich micro-colonies. Antimicrob. Agents Chemother..

[35] Buckley H.L., Hart-Cooper W.M., Kim J.H., Faulkner D.M., Cheng L.W., Chan K.L., Vulpe C.D., Orts W.J., Amrose S.E., Mulvihill M.J. (2017). Design and testing of safer, more effective preservatives for consumer products. ACS Sustain. Chem. Eng..

[36] Kim J.H., Mahoney N., Chan K.L., Campbell B.C., Haff R.P., Stanker L.H. (2014). Use of benzo analogs to enhance antimycotic activity of kresoxim methyl for control of aflatoxigenic fungal pathogens. Front. Microbiol..

[37] Bisogno F., Mascoti L., Sanchez C., Garibotto F., Giannini F., Kurina-Sanz M., Enriz R. (2007). Structure-antifungal activity relationship of cinnamic acid derivatives. J. Agric. Food Chem..

[38] Tawata S., Taira S., Kobamoto N., Zhu J., Ishihara M., Toyama S. (1996). Synthesis and antifungal activity of cinnamic acid esters. Biosci. Biotechnol. Biochem..

[39] Zhou K., Chen D., Li B., Zhang B., Miao F., Zhou L. (2017). Bioactivity and structure-activity relationship of cinnamic acid esters and their derivatives as potential antifungal agents for plant protection. PLoS ONE.

[40] Odds F.C. (2003). Synergy, antagonism, and what the chequerboard puts between them. J. Antimicrob. Chemother..

[41] Kirkman T.W. Statistics to Use. http://www.physics.csbsju.edu/stats/.

