# Supplementary material for: Cinnamic Acid Analogs as Intervention Catalysts for Overcoming Antifungal Tolerance
Source: Molecules. 2017 Oct 21;22(10):1783. doi: 10.3390/molecules22101783 (PMC6151797; doi:10.3390/molecules22101783)
Supplement: Supplementary file 1 [file molecules-22-01783-s001.pdf]

Supplementary Figures

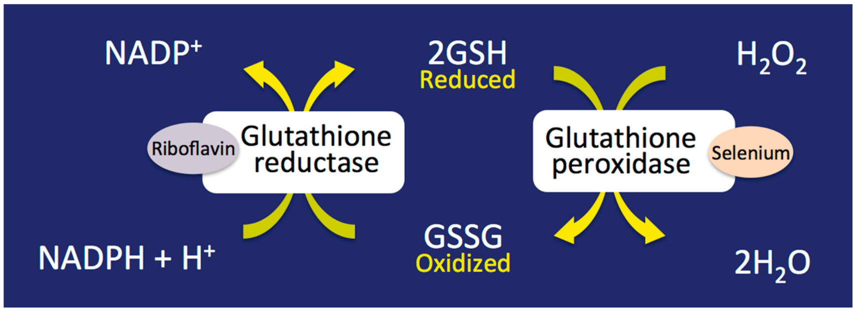

Figure S1. Diagram showing the role of glutathione reductase to reduce GSSG (Oxidized glutathione) to GSH (Reduced glutathione) (Adapted from [21]).

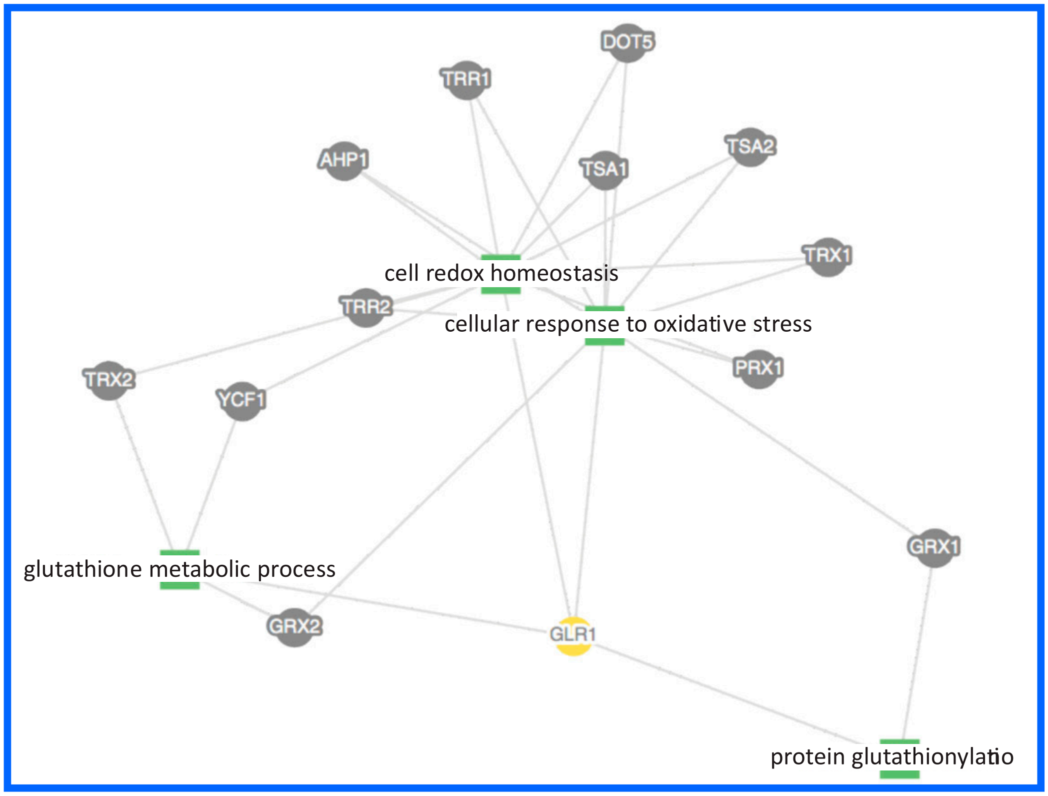

Figure S2. Diagram showing *GLR1* interacting genes. Functions of *GLR1* interacting genes include: (1) Cell redox homeostasis, (2) Cellular response to oxidative stress, (3) Protein glutathionylation, and (4) Glutathione metabolic process (Downloaded from *Saccharomyces cerevisiae* Genome Database; [22]).
